# Chilaiditi’s Sign: A Case Report

**DOI:** 10.7759/cureus.6230

**Published:** 2019-11-25

**Authors:** Raman J Sohal, Steven H Adams, Vishal Phogat, Ceren Durer, Abha Harish

**Affiliations:** 1 Medicine, SUNY Upstate Medical University, Syracuse, USA; 2 General Medicine, S.G.T. Medical College, Hospital & Research Institute, Gurgaon, IND; 3 Internal Medicine, SUNY Upstate Medical University, Syracuse, USA

**Keywords:** chilaiditi's sign, pneumoperitoneum, syndrome, abdominal pain, surgical intervention

## Abstract

Chilaiditi’s sign refers to the interposition of the colon (usually the transverse colon) between the diaphragm and the liver. When associated with abdominal pain it is referred to as Chilaiditi’s syndrome. Chilaiditi’s sign is rare entity with an estimated incidence of 0.025 to 0.28% worldwide. The sign occurs more frequently in males, with a male to female ratio of 4:1. Apparent pneumoperitoneum seen on imaging below the right hemidiaphragm, a life-threatening condition, may in fact be merely Chilaiditi’s sign. Awareness of this phenomenon and its consideration as a differential diagnosis is essential to prevent unnecessary laparoscopic intervention.

Here we present a case of a 74-year-old male who was incidentally found to have free air under the diaphragm without symptoms of abdominal pain. After further evaluation by the radiologists and surgeons it was concluded that he had Chilaiditi’s sign and no further intervention was required. However, due to the lack of awareness of this radiographic finding patients can be subjected to unnecessary surgical intervention.

## Introduction

Chilaiditi’s sign refers to the interposition of the colon (usually the transverse colon) between the diaphragm and the liver. When associated with abdominal pain it is referred to as Chilaiditi’s syndrome. Chilaiditi’s sign is uncommon with an estimated incidence of 0.025 to 0.28% worldwide [1]. The sign occurs more frequently in males, with a male to female ratio of 4:1 [2]. Apparent pneumoperitoneum seen on imaging below the right hemidiaphragm, a life-threatening condition, may in fact be merely Chilaiditi’s sign. Awareness of this phenomenon and its consideration as a differential diagnosis is essential to prevent unnecessary laparoscopic intervention [1-3].

## Case presentation

A 74-year-old male came to the ED with the acute onset of worsening back pain and lower extremity weakness. His past medical history included chronic lower back pain from severe spinal stenosis, hypothyroidism, hypertension, hyperlipidemia, anxiety, benign prostatic hyperplasia, and a pulmonary nodule. Upon review of symptoms, he was found to have chills and decreased activity. Initially, there was concern for cord compression and epidural abscess. He denied any history of fever, nausea, vomiting, or abdominal pain at admission. He reported no history of recent changes in his bowel or bladder habits. He described having had dental work performed two weeks prior to presentation.

CT spine showed severe degenerative lumbar spine along with multilevel cervical spine stenosis. Neurosurgery was consulted who recommended MRI. Preliminary blood cultures grew gram positive cocci in clusters and the patient was started on vancomycin plus cefazolin. ID team was consulted and they recommended to continue with vancomycin and cefazolin until final blood culture report. Repeat blood culture grew MSSA. The source of infection was thought to be abscess in spine, however, MRI cervical/thoracic/lumbar spine showed no evidence of osteomyelitis, epidural abscess or cord compression. Yet, it showed multilevel severe spinal canal stenosis worse at L4-L5 along with multilevel bilateral neuroforaminal stenosis. Neurosurgery recommended only pain management and no acute intervention stating the surgery can be done in the outpatient setting. The patient was started on intravenous oxacillin 2 gram every four hours for total of four weeks of therapy.

A transesophageal echo was obtained which showed small 4-mm linear echo density on the ventricular side of the left coronary cusp of the aortic valve. Differentials included Lambl’s excrescence versus valvular vegetation. Cardiovascular surgery recommended against the intervention and to continue with conservative measures; antibiotics and pain management. The source of the patient’s MSSA bacteremia was deemed to be secondary to infective endocarditis.

**Figure 1:**
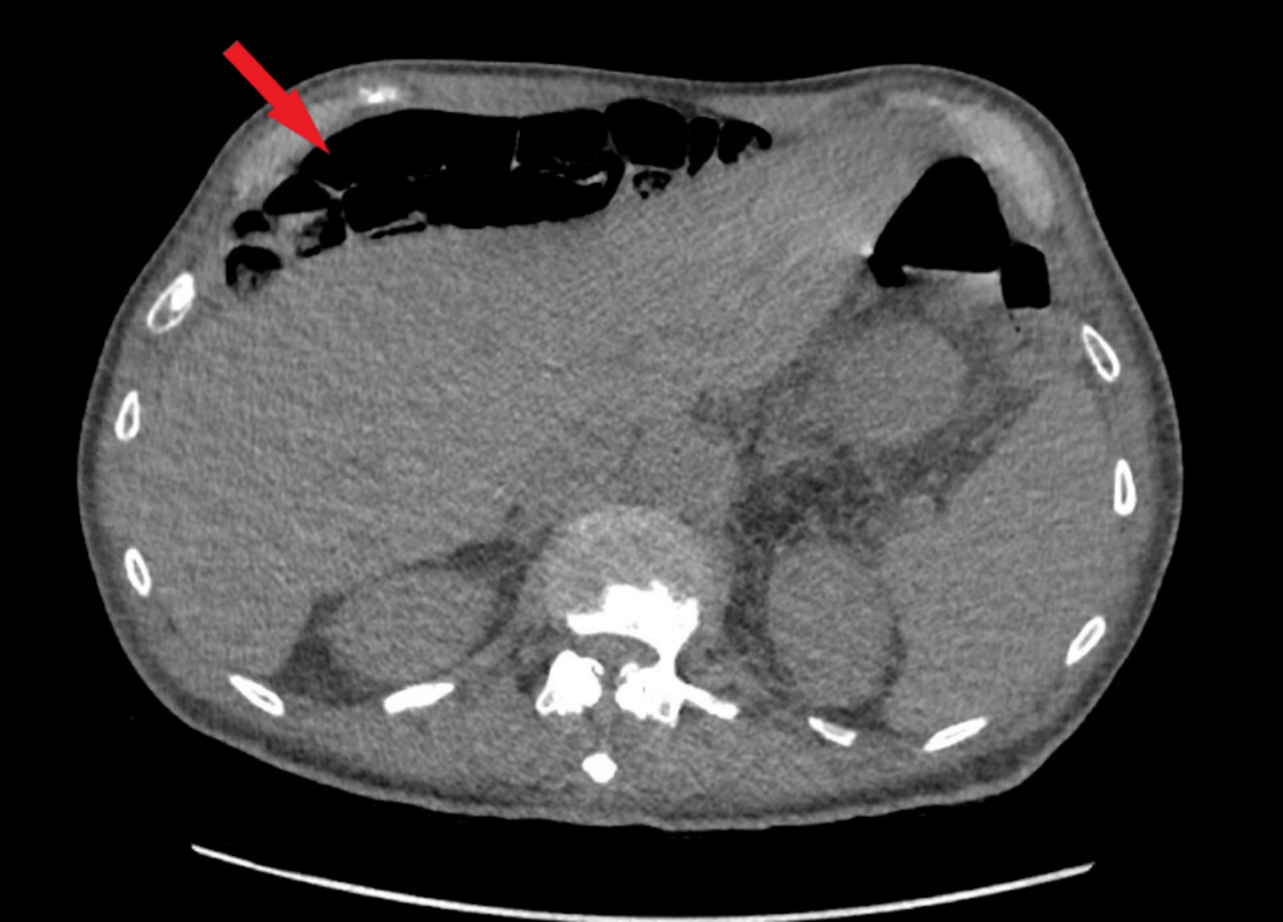
CT of the abdomen. Red arrow shows the interposition of bowel loops anterior to the liver.

**Figure 2:**
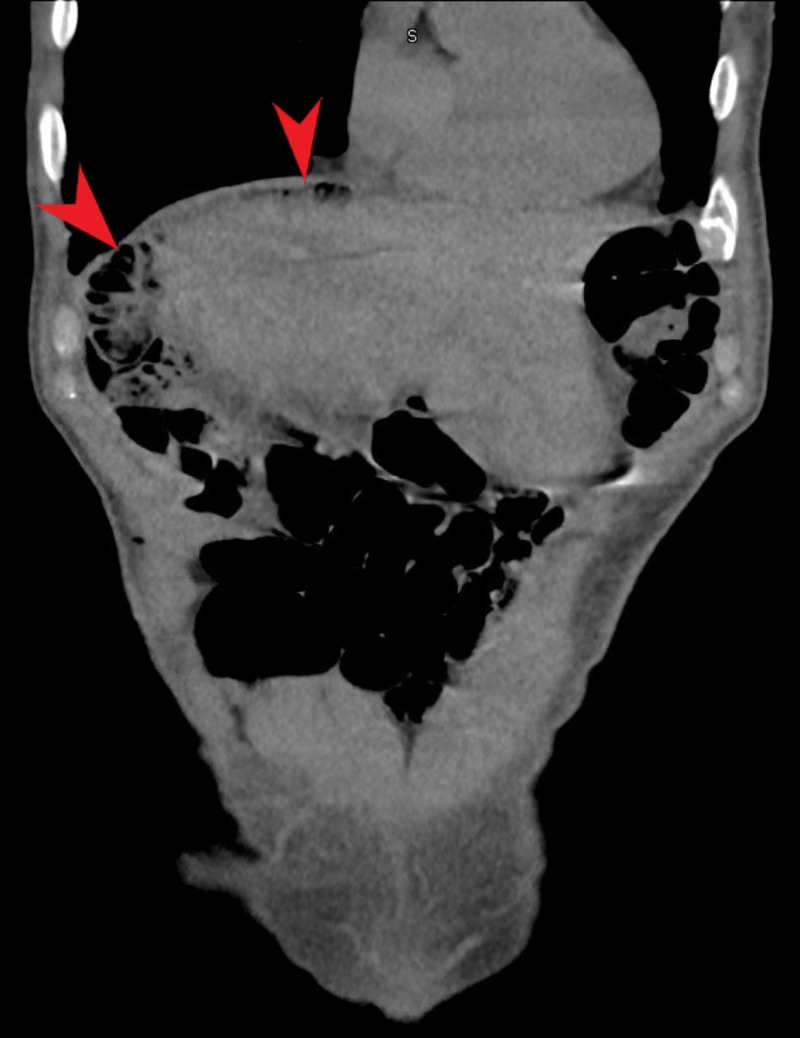
CT of the abdomen. Red arrows show loops of bowel in the right subdiaphragmatic space.

A CT chest was performed inpatient to further elucidate the pulmonary nodule. During this exam CT chest showed bubbles of gas in the upper abdomen, rendered likely intraluminal by the radiologist’s report. The CT chest finding was followed up with a CT abdomen to rule out pneumoperitoneum. CT abdomen reported Chilaiditi sign with anterior interposition of the colon to the liver (see Figures 1-2). No bowel perforation, small bowel obstruction, or surrounding inflammatory changes were noted on CT imaging.

## Discussion

Chilaiditi’s sign is the malposition of the colon between the diaphragm and the liver. Thus, it is the manipulation of any of these three organs (the diaphragm, liver, or colon) which can predispose to such organ misplacement. The diaphragmatic causes include any factor that raises the diaphragm, thereby increasing the space between it and the liver. These include phrenic nerve palsy which produces a right-sided diaphragmatic hemiparesis, and the congenital loss of the muscular fibers of the diaphragm which can result in diaphragmatic eventration [3]. Potential hepatic causes of Chilaiditi’s sign are the loss of tone in the falciform ligament, which normally functions to connect the liver to the anterior abdominal wall and diaphragm, or a small liver secondary to cirrhosis or hepatectomy [4]. Dolichocolon, an abnormally long large intestine, can also be a cause of Chilaiditi’s sign as the extra colon finds home in random spaces [5]. Ascites and obesity have also been associated with increased risk of developing Chilaiditi’s sign [4,6]. Additionally, patients with abdominal adhesions are at risk. These fibrous tissue bands, if formed between the correct organs, may facilitate the colon’s interposition between the liver and diaphragm [7].

Symptomatic patients will frequently present with nausea, vomiting, and loss of appetite [8]. Cases of Chilaiditi’s sign causing severe dyspnea have also been reported [9-11]. A diagnosis of Chilaiditi's is based on imaging with techniques including chest or abdominal X-rays, CT scanning, or sonography [12]. In some cases, such as with our patient, more than one imaging series or modality may be necessary to make the diagnosis with certainty [5]. If symptomatic and left untreated, severe complications include intestinal obstruction, bowel wall ischemia and perforation, and respiratory failure. However, conservative treatment including bed rest, IV fluids, endoscopic bowel decompression, and laxatives is often sufficient [13]. Surgical intervention should be considered in patients who show no improvement with conservative measures and those with serious complications [14].

Chilaiditi’s sign in our patient was an incidental finding on a CT thorax. His presentation fit with the epidemiology of the disease described in the literature - elderly males [6]. However, he did not appear to have any of the risk factors, such as cirrhosis, ascites, obesity, a history of abdominal surgery causing adhesions, or diaphragmatic palsy, that could predispose him to the interposition of intestine between liver and diaphragm. This atypical finding was concerning and led to a follow-up CT abdomen and pelvis which revealed no pneumoperitoneum, bowel obstruction or perforation. Per hospital protocol, the surgical service was consulted and based upon their clinical evaluation of the patient a recommendation was made to continue with conservative management.

## Conclusions

Chilaiditi syndrome is relatively uncommon and is easily misdiagnosed. This case highlights the importance of clinical awareness of this sign among physicians to reduce the need for unnecessary surgical intervention.
